# Gene and Cell Therapy for Muscular Dystrophies: Are We Getting There?

**DOI:** 10.1089/hum.2018.151

**Published:** 2018-10-12

**Authors:** Francesco Galli, Laricia Bragg, Linda Meggiolaro, Maira Rossi, Miriam Caffarini, Naila Naz, Sabrina Santoleri, Giulio Cossu

**Affiliations:** Division of Cell Matrix Biology and Regenerative Medicine, University of Manchester, Manchester, United Kingdom.

**Keywords:** gene therapy, cell therapy, muscular dystrophy

## Abstract

In the last few years, significant advances have occurred in the preclinical and clinical work toward gene and cell therapy for muscular dystrophy. At the time of this writing, several trials are ongoing and more are expected to start. It is thus a time of expectation, even though many hurdles remain and it is unclear whether they will be overcome with current strategies or if further improvements will be necessary.

## Introduction

Muscular dystrophies are a group of genetic diseases characterized by progressive wasting of skeletal and often cardiac muscle. This wasting compromises patient mobility and subsequently respiratory and cardiac functions, leading to wheelchair dependency, respiratory and/or cardiac failure, and premature death.^1^ Mutations often affect genes that encode proteins forming a complex that links the cytoskeleton and the basal lamina. In the absence of one of these proteins, the whole complex is disrupted, which leads to increased fragility of the sarcolemma. This fragility in turn results in increased calcium entry and focal or diffuse damage to the fiber during contraction, especially eccentric contraction, such as stepping down a stair.^2^ Damaged or dead fibers are initially repaired or replaced by satellite cells, up to 20 years ago the only known myogenic cell present in postnatal life.^3^ However, the newly regenerated fibers share the same molecular defect and produce fibers that are also prone to degeneration. The various forms of muscular dystrophy differ greatly in severity, which correlates with when the majority of fibers are lost and muscle tissue is progressively replaced by connective and adipose tissue. The general consensus is that all therapies will be ineffective at this late stage; therefore, any intervention should be carried out as soon as possible, before the disease compromises tissue integrity.

Currently, steroids represent the only standard therapy for dystrophic patients, but they only delay the progression of the disease and have serious side effects. Many novel therapeutic approaches have entered clinical experimentation with encouraging results, but none have yet reached significant and long-lasting clinical efficacy.^4^ These include new drugs, gene therapy, exon skipping, PTC124 (which triggers premature STOP-codon read-through), and cell therapy.

In this review we focus on current advances in gene and cell therapy, briefly describing the most recent work, comparing the benefits and drawbacks of the two strategies, noting the remaining bottlenecks, and speculating on how they may be solved in the near future. There are obvious differences but also similarities between cell and gene therapy, especially considering that *ex vivo* gene therapy is a field bridging the two, with the majority of successes having so far been reported for other genetic diseases.

## Gene Therapy

In this section we will briefly discuss clinical and preclinical work of *in vivo* gene therapy, in which the vector is directly injected into the patient tissues and organs. The basic concept of this approach is simple: the vector carries either a wild-type (wt) copy of the mutated gene (replacement) or molecules that repair the DNA or the mRNA into the diseased cell, leading to the production of a normal or quasi-normal protein at a sufficient level to carry out its specific function. Obviously, the level varies between enzymes, for which a few percentage of the normal level is usually sufficient to do the job, and structural proteins, such as dystrophin, for which it has been indirectly calculated that 20%–30% of the normal level is the minimum level necessary to restore function.

The first choice concerns the vector, and adeno-associated vectors (AAVs) are currently center-stage in gene therapy for muscular dystrophies as for most genetic diseases.^5^ Around the end of last century, early attempts using adenovectors initially raised excitement when tested in newborn mice, but they were abandoned because their large size would prevent crossing a mature basal lamina around the muscle fiber and because of a strong immune reaction that had not been apparent in neonatal animals.^6^ Other vectors, such as herpes-derived vectors, have also been tried, but they never progressed to clinical experimentation.^7^ This situation has also been the case also for nonviral vector so far, mainly because of their low efficiency, although new generations of these molecules raise some hope.^8^

AAVs are small, which is beneficial in terms of their diffusion into tissues but a drawback in terms of their capacity. Owing to their small size, they can only accommodate relatively small cDNA, up to 5 kb, clearly not enough for cDNA encoding large proteins such as dystrophin, utrophin, or laminin. Several laboratories have worked for many years, starting with the observation of a large in-frame deletion of the dystrophin gene in patients with Becker muscular dystrophy (the milder form of Duchenne muscular dystrophy [DMD]) who were able to carry on an almost normal life. Mini and micro dystrophin have been progressively optimized, and the currently available version appears to have the right size to be accommodated in an AAV, while largely maintaining all or most domains needed to exert the protein function.^5^

A second problem is represented by the immune response of the host to the AAV capsid proteins and to the gene products eventually expressed by the vector.^9^ There are many different serotypes of AAVs, indicated by a progressive number, often with a specific tropism for one or more tissues (AAV2 and 9 being the ones of choice for skeletal and cardiac muscle). It has been calculated that approximately half of the human population has been exposed to one or more serotypes of the corresponding natural virus. Consequently, patients need to undergo preliminary screening to ensure that pre-existing neutralizing antibodies do not prevent any effect of the vector. Even in patients not previously exposed to a given serotype, the first administration of the vector induces an immune response that apparently does not attack cells already transduced, probably because of the progressive disappearance of the viral antigens during the weeks needed to mount the immune response. However, a second administration of the same serotype would be ineffective. Selecting a different serotype for a second administration and/or treating the host with immune modulatory drugs to blunt the immune response during the period of vector administration may address this issue. Whether these strategies may confer long time escape from the immune system remains to be seen. In addition, it has long been considered that the immune system may never have encountered the gene product, or part of it, and thus it may also elicit an immune response. In the case of dystrophin, clinical observation has shown a large and partly unexplained variability, with some patients immunized against dystrophin, sometimes even before the gene therapy, and others who do not have and do not mount an immune response.^10^ When gene correction rather than replacement is pursued, the correcting molecule may be an RNA or a protein and the latter may be an antigen, especially if not human, or even worse, bacterial.

A third problem is represented by the nature of the AAV because it usually does not integrate into the host cell genome and therefore is progressively lost from rapidly dividing cells. This problem is not a particular concern for striated muscle, in which the differentiated cells do not divide any longer and in principle last for the life of the host. Indeed, persistence of the vector DNA has been observed over a period of years in different preclinical models, but it requires that, once corrected with AAV, the dystrophic muscle fibers and cardiomyocytes become “normal” and thus long-lived because the vector DNA would be lost with the dying cell.^11^

**Figure 1.**
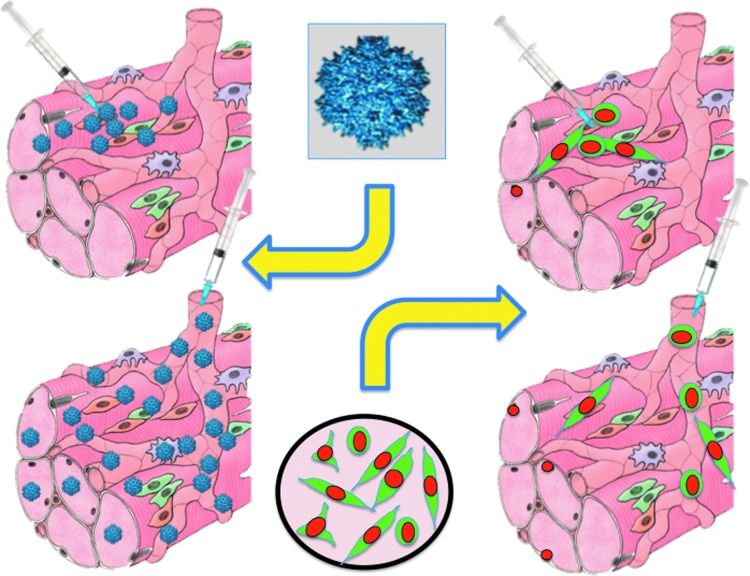
Similarity of delivery routes for adeno-associated vectors (*left*) or cells (*right*). In both cases, local transplantation (*top*) leads to a high concentration but only in the side of injection. In contrast vascular delivery (*bottom*) leads to widespread concentration, which however may not be sufficient for a significant therapeutic effect. The figure was produced using Servier Medical Art (www.servier.com).

The fourth and probably most complex problem is delivery (Fig. 1). Delivery depends on the route of administration and, most importantly, on the anatomy of the target tissue. In the case of the retina or the substantia nigra, direct delivery may be achieved by injecting the vector directly into or near the target.^12,13^ For a tissue like skeletal muscle, which is distributed throughout the body, a systemic route of delivery is necessary and the predominant one has been intravenous (IV). Although an intra-arterial catheterization is possible and would avoid capillary filters such as the liver or the lung, IV injection has been the preferred route of administration so far, most likely for its simplicity. Other routes are possible in principle, such as intraperitoneal or subcutaneous, but they have not been used to any significant extent, probably because diffusion of the vector would have been more difficult. Of course an intramuscular injection is the simplest route of administration, but it only targets the area where the vector would diffuse. Consequently, this route is used as a proof of principle for vector activity and immunogenicity, but it is not informative on systemic distribution. In preclinical studies in the dog, a loco-regional distribution to a limb was only achieved by trans-venous injection with a tourniquet applied to enhance local pressure and consequently cause extravasation of the vector.^14^ In subsequent studies, systemic IV delivery was also found to be sufficient, but the reason for this difference with the initial studies remains unclear.^15,16^ Obviously, the loco-regional delivery would not allow targeting of the diaphragm or the heart, and thus systemic IV delivery is certainly preferable because it is simpler and apparently able to achieve wider vector distribution.

Even when these problems are solved, the complexity and costs associated with the production of large amounts of AAVs will remain as a significant hurdle.^17^ Most data concur that very high titers (10^13^ to 10^14^ vector genome/kg) are needed to achieve significant dystrophin expression. To get enough GMP-grade vector for a child is a challenge for most facilities, and it would be even more difficult for adults affected by milder forms of muscular dystrophy. However, it is probable that, in case of clinical efficacy, new advances in the field will lead to optimization of vector production and consequently a reduction in costs.

Finally, the method of gene correction is still debated in light of previous failures and novel perspectives. By simplifying this complex scenario, it may be stated that gene replacement remains the first choice at the moment, though the choice may be different in the future. Many versions of micro-dystrophin have given different results in mice and dogs, with some offering protection from the disease, especially if treatment started early.^16,18^ New, optimized versions of micro-dystrophin, including functional domains are now available.^19^ In addition, four clinical trials have been started, which are also supported by spectacular efficacy and lack of toxicity of AAV systemically delivered to patients with spinal muscular atrophy. The AAV accommodates the full-length cDNA of the mutated gene, however. At the time of writing, a press release from Sarepta has announced efficacy and safety in the first three DMD patients treated.^*^ However, a recent study in primates and piglets showed significant liver and neural toxicity, respectively, which required euthanasia for many treated animals. For a more in-depth discussion of these topics, a recent excellent review (Duan, 2018) is recommended.^20^ Other correction strategies rely on AAV that delivered small nuclear RNA, such as U7, that can skip the mutated exon to restore the reading frame and produce a shorter but functional dystrophin.^14,21^ This strategy is the same that as that achieved through systemic administration of oligonucleotides, which has been widely tested in several trials with different oligo backbones (see below). Finally, AAV may deliver enzymes that edit the genome to permanently repair the damage. Currently, the most popular is certainly CRISPR-Cas9, which appears to cut the mutated region and eventually replace it with the correct sequence.^22,23^ The current enthusiasm for this enzyme does not overlook the fact that Cas9 is a bacterial protein that is potentially highly immunogenic and may work well *in vitro* (see below), while being likely to induce a strong immune reaction *in vivo*. Transient immune suppression or a new version of the enzyme, ideally short lived to reduce the time of exposure to the immune system, may help to solve this problem. Possible Cas9 toxicity involving p53 is described in detail in the next section; it would be less problematic for post-mitotic nuclei but still remains an issue.^24,25^ Despite significant open issues, this is an exciting period for gene therapy of muscular dystrophies, with promising, albeit preliminary results that may finally lead to an efficacious therapy after decades of hopes and disillusionment.

Halfway between gene therapy and conventional drugs stand oligonucleotide-mediated exon skipping and PTC124. They are not gene therapy *sensu strictu* because the therapeutic agent is not delivered by a vector, but rather a new generation of molecular drugs that correct the genetic defect. These strategies have reached marketing authorization but have not yet shown conclusive evidence of significant and long-lasting clinical efficacy. There are many recent reviews, and thus we will only provide a short overview, referring the reader to these reviews for more extensive coverage of the topics.^26–30^ PTC124 (ataluren) is a derivative of gentamycin, allowing the transcriptional machinery to skip premature termination code and produce a shorter but still functional version of dystrophin. After much preclinical and clinical work, a recent phase III trial has demonstrated that it has modest efficacy only in patients with partially preserved walking ability.^31^ This outcome is in a sense expected because muscles that are too deteriorated cannot be rescued when there are too few fibers to produce dystrophin, while in patients who are still walking well, the effect is not (yet) visible. Therefore, treatment should be continued until the placebo group begins to deteriorate significantly. Oligonucleotides bind to and mask the acceptor and donor splice sites of the exon containing the mutation, thus allowing production of a quasi-dystrophin, missing only one or a few exons. Also, oligos produce a striking correction *in vitro* but are much less effective *in vivo*, especially in large animals, most likely because of diffusion problems with a large molecule that are obviously exacerbated in a fibrotic muscle. For these, like for all strategies, a general consensus exists that treatment should start as early as possible, ideally at diagnosis, before pathological changes occur in the dystrophic muscle. The problem is that in this case, treatment should last for years, until placebo control groups begin to show a decline in motor function.

## Cell Therapy

At variance with gene therapy, cell therapy is less popular now because the hurdles preventing clinical efficacy, although similar to those in gene therapy, appear to be more difficult to overcome. Moreover, while the same AAV promises to treat both skeletal and cardiac muscle, essentially two birds with a stone, it is highly unlikely that the same cell type will be applicable for both skeletal and cardiac muscle, for which cell therapy trials for other pathologies have met very limited or no efficacy.^32^ Several issues must be considered for cell therapy. The first concerns the ideal cell type. A robust potency to differentiate into skeletal muscle cells is currently recognized as a necessary, although not sufficient feature to be tested in preclinical models and eventually clinically. For other cell types, such as bone marrow, fibroblasts, and mesenchymal stromal or adipose-derived cells, the evidence for spontaneous skeletal myogenesis is scant. Thus, some tool, such as an inducible expression of MyoD, would be necessary.

In terms of myogenesis, satellite cells are definitely the first choice. They are considered the “bona fide” stem cell of skeletal muscle, responsible for both postnatal muscle growth and regeneration. First described by Mauro,^3^ satellite cells are currently intensely studied for their role in regeneration and senescence. Even though serial transplantation experiments showed their “stemness” in the mouse, human cells have limited self-renewal ability, as demonstrated by more severe forms of muscular dystrophies in which repeated degeneration/regeneration cycles cause their exhaustion in a few years.^33^

Following successful transplantation of mouse satellite cells in a mouse muscle,^34^ few clinical trials of cell transplantation have been carried out for DMD. After *in vitro* expansion, satellite cell–derived myogenic progenitors were directly injected in the muscles of a few patients, with no toxicity or efficacy. Failure was due to massive death of transplanted cells. In addition, transplanted cells remained in the area of injection, which would make it difficult to achieve an even distribution within the whole muscle. For large muscles, intra-muscular transplantation would be extremely challenging if possible at all. For reviews see.^35,36^

Nevertheless, after many years of work aimed at optimizing the intramuscular transplantation protocol, this approach has now entered clinical experimentation for localized forms such as oculo-pharyngeal muscular dystrophy.^37^ Satellite cells derived from nonaffected muscle of the same patients, although genetically noncorrected, were expanded *in vitro* and then injected in the pharyngeal muscles, where they ameliorated swallowing, the main problem in this form of muscular dystrophy. Other clinical trials using satellite cell–derived progenitors are ongoing for local damage to muscle, for example, damage underlying sphincter incontinence.^38^

From existing research, we can conclude that intramuscular injection of satellite cells seems to be the therapy of choice for localized forms of muscular dystrophy or other muscle diseases, but not for forms affecting most of the body muscles (Fig. 1). Indeed, delivery is the second major problem for cell therapy. It was long considered that systemic injection of myogenic cells would overcome this hurdle. However, once satellite cells enter the circulation, extravasation is not possible and they simply accumulate in the capillaries as micro-thrombi. In contrast, widespread distribution would be possible by using blood-borne stem/progenitor cells. This perspective became theoretically possible in the late 1990s, with the demonstration of cells in the bone marrow that could contribute to muscle regeneration upon bone marrow transplantation.^39^ However, bone marrow transplantation failed to restore dystrophin expression in a significant fraction of muscle fibers in the receiving animal.^40^

During the following years, several types of mesoderm stem/progenitor cells were isolated from bone marrow, fat, skin, and vessels, some of which appeared to possess the properties needed for a successful cell therapy approach: simple isolation and the ability to proliferate *in vitro*, cross the vessel wall when delivered in the circulation, and differentiate efficiently *in vitro* and *in vivo* into skeletal muscle fibers.^36^ Skeletal muscle contains many other cell types beside satellite cells that were initially identified by classic histology (fibroblasts, vessel pericytes and smooth muscle, endothelium, Schwann cells, and tenocytes) and more recently by the expression of specific markers such as Pw1 for PW1(+)/Pax7(-) interstitial cells or PDGF receptor alpha for fibro-adipogenic progenitors.^41,42^ None of these cells has been characterized as a stem cell and likely they are not, but some may participate in muscle growth or regeneration.

Mesoangioblasts are *in vitro* counterparts of muscle perivascular cells that express tissue nonspecific alkaline phosphatase (*TNAP*).^43^ Through the use of a TNAP-cre mouse, they were shown to contribute to muscle growth and regeneration to a minor extent.^44^ Human donor mesoangioblasts, derived from an HLA-matched brother, were intra-arterially transplanted in five DMD patients via their femoral and subclavian arteries (because of the cells' ability to cross the inflamed vessel wall) in four consecutive infusions at increasing cell doses. The trial showed safety but lacked clear clinical efficacy, although low but unequivocal expression of donor dystrophin was detected in the youngest patient. Several reasons explains the low efficacy, among which the most important was the very low engraftment with only 0.7% of donor DNA detected in the biopsy of the same patient.^45^ This observation leads to the probably most difficult hurdle: low engraftment.

Recent results in cell therapy have confirmed that success occurs in diseases of tissues such as the blood or the epithelia, in which diseased cells can be ablated, thus creating “space” for donor cells.^46^ In diseases affecting other tissues/organs, engraftment will be unavoidably low and thus alternative strategies are needed. For metachromatic leukodystrophy, overexpression of the wt copy of the mutated enzyme, arylsulfatase, was achieved by lentivector-mediated transduction of patients' own hematopoietic stem cells (HSCs). HSC-derived microglia overexpressed and released the enzyme that was taken up by neurons, thus preventing their death. This approach led to complete prevention of the disease in children treated at a very early age.^47^

Being a multinucleated cell, the muscle fiber offers a different opportunity: it is indeed possible to engineer donor cells as “Trojan horses” that may enter regenerating fibers and then also correct neighboring resident nuclei. From this perspective, overexpression of microdystrophins or sarcoglycan, ideally from a strong muscle-specific promoter may be a possibility. Alternatively, the role of exosomes in skeletal muscle regeneration may be exploited for therapeutic purposes as suggested by preliminary but encouraging data.

It was recently shown that MSC exosomes promote dystrophic muscle regeneration.^48^ Moreover, a recent report suggests that exosomes secreted by cardiosphere-derived cells (CDCs) transiently restore partial expression of full-length dystrophin in *mdx* mice, reproducing the benefits of CDCs.^49^ All these novel and exciting results await confirmation by independent laboratories.

In all *ex vivo* gene therapy protocols, lentivectors are now the vectors of choice because they show a much lower risk of insertional mutagenesis in comparison with the retroviral vector (derived from murine Moloney Leukemia Virus) used in the first trials. This lower risk is due to the fact that lentiviruses integrate randomly in the genome, while retroviruses select for transcriptionally active regions, thus increasing the possibility of activating “dangerous” genes.^50^ However, if engraftment is low, it remains to be seen whether overexpression from few cells is sufficient to compensate for the large majority of nuclei that cannot synthesize the missing protein. This possibility is especially important because a structural protein is certainly needed at a higher cellular concentration in comparison with an enzyme.

We are currently pursuing a different strategy: by transducing donor myogenic cells with a lentivector expressing the U7 snRNA, engineered to skip exon 51, we plan to exploit the diffusion of snRNA to neighboring nuclei and induce exon skipping and thus amplify the production of dystrophin. Other strategies may be and possibly are being similarly pursued. For example, transient expression of CRISPR Cas9 from a non-integrating lentivector, may genetically correct also neighboring nuclei, thus amplifying the genetic correction. The advantage over exon skipping would be the permanent correction of the genetic defect, but the risk would be the induction of an immune response that may or not be controlled by immune suppression.

In recent years, induced pluripotent stem cells (iPSCs) have raised much hope.^51^ Adult cells from the patient can be reprogrammed with the Yamanaka factors to a stage comparable with bona fide embryonic stem cells. The cells can be genetically corrected, ideally in this case with CRISPR Cas9 for a definitive effect, and then differentiated to muscle progenitor cells. If the use of Cas9 occurs *ex vivo*, there is no risk of an immune reaction and, in the case of iPSCs, clones with appropriate correction and genome integrity may be selected before induction of differentiation into the desired cell type. This protocol would result in unlimited number of autologous, nonimmunogenic myogenic progenitors whose genome is now “normal” (*i.e.*, the mutation has been erased from it).

Still, the problems of delivery and engraftment remain. While the first may be addressed by differentiating iPSCs to mesoangioblasts, engraftment would remain low and the genetically corrected nucleus would still have to produce enough dystrophin for the resident noncorrected nuclei.^52^ Moreover, two very recent papers showed that the high efficiency of double-strand breaks induced by Cas9 induce a p53-mediated DNA damage response and cell cycle arrest in iPSCs and immortalized cells.^24,25^ Thus, p53 inhibition may improve genome editing and survival and proliferation of edited cells. However, the selection of p53-mutated cells is a major concern for safety because p53 inhibition could increase the risk of cancer in a short and long time period. Whether the same holds true for other cells remains to be seen. Table 1 summarizes current features of cell and gene therapy strategies.

**Table 1.** A synoptic view of current issues in gene and cell therapy

*Challenge*	In vivo *gene therapy*	Ex vivo *gene therapy*	*Cell therapy*
Vector of choice	Adeno-associated vector	Lentivector	—
Cell of choice	—	Satellite cells	Satellite cells Mesoangioblasts
		Mesoangioblasts	Induced pluripotent stem cells
		Induced pluripotent stem cells	
Delivery route	Intramuscular	Intramuscular	Intramuscular
	Intravenous	Intra-arterial	Intra-arterial
	Intravenous loco-regional	Intravenous	Intravenous
Gene correction	Replacement	Replacement	Replacement
	Exon skipping	Exon skipping	
	Genome editing	Genome editing	
Immune response	Wild-type gene product	Wild-type gene product	Wild-type gene product
	Therapeutic protein (*e.g.*, nuclease)	Therapeutic protein (*e.g.*, nuclease)	
	Vector (capsid)		

The table offers a simple overview of current major issues in the field.

## Conclusions and future perspectives

This brief survey of the reported work in gene and cell therapy shows that both hold promise and face problems, but gene therapy is at least one step ahead and closer to clinical applications. This position may be due to the relatively more straightforward strategy and the fact that a vector may be useable for all patients, or at least for all patients with the same mutation and immune status, while cell therapy is an extreme form of personalized medicine, in which one medicinal product is produced and administered to one patient only. As a consequence, industry interest has been more focused on gene therapy, which has received much more financial support and investment than cell therapy.

However, for gene therapy, the following questions need definitive answers: Will safety be confirmed in large cohorts of patients? How long will the vector DNA and thus a sufficient level of the gene product persist in a growing muscle? Will a re-administration be possible, should it become necessary? How can the immune system be controlled at short and long term? How routine and affordable may GMP vector production become? When these questions are answered, then a real cure for DMD and later other forms of muscular dystrophy may finally be achieved after decades of frustrating attempts.

And what about cell therapy? In a commentary we wrote 18 years ago, we wondered whether cell therapy was a real opportunity or simply “wishful thinking.”^53^After all these years, there are still no definitive answers, but reasons exist for continuing on this route. Low engraftment may be compensated by trans-correction in multinucleated muscle fibers, immune problems are much lower than with gene therapy, and in principle, one corrected muscle fiber will last for the patient's lifespan, with a much reduced chance of loss of the therapeutic agent. However, no one knows what tomorrow brings, and we will have to wait and see the outcome of the ongoing trials to reach a more definitive conclusion.

## Notes

^*^ http://strongly.mda.org/sarepta-reports-positive-preliminary-findings-in-dmd-gene-therapy-trial/ (last accessed September 12, 2018).
